# Perceived Organizational Support and Workplace Conflict: The Mediating Role of Failure-Related Trust

**DOI:** 10.3389/fpsyg.2018.02704

**Published:** 2019-01-09

**Authors:** Gaëtane Caesens, Florence Stinglhamber, Stéphanie Demoulin, Matthias De Wilde, Adrien Mierop

**Affiliations:** Psychological Sciences Research Institute, Université catholique de Louvain, Louvain-la-Neuve, Belgium

**Keywords:** perceived organizational support, workplace conflict, relationship conflict, task conflict, failure-related trust

## Abstract

The aim of the present research was twofold. First, we examined the effects of perceived organizational support (POS) on workplace conflict (i.e., relationship conflict and task conflict). Second, we identified one mechanism explaining these relationships, namely failure-related trust. Using a sample of 263 teachers from Belgium, the results of Study 1 indicated that POS is negatively related to relationship conflict and is also, unexpectedly, negatively related to task conflict. Furthermore, using a sample of 477 Belgian employees, Study 2 replicated these results and further demonstrated that failure-related trust mediates the negative relationship between POS and both types of workplace conflict. Theoretical and practical insights of this research are discussed.

## Introduction

Workplace conflict plays a critical role in the organizational life because of its important consequences for both organizations (e.g., performance; e.g., Meier et al., 2013) and individuals (e.g., well-being; e.g., Sonnentag et al., 2013). For these reasons, this construct has received a vivid interest from scholars in the management literature (e.g., De Dreu and Weingart, 2003; Lu et al., 2011; Sonnentag et al., 2013). Scholars have drawn a distinction in the literature between relationship and task conflict (e.g., Pinkley, 1991; Jehn, 1995; Amason, 1996). *Relationship conflict* refers to interpersonal incompatibilities involving feelings of tension and friction, whereas *task conflict* refers to disagreements between individuals “about the correct way to solve a problem” (Danielsson et al., 2015, p. 144). Given the potential benefits of task conflict in terms of increasing divergent ideas (e.g., Amason, 1996) or innovative behaviors (Lu et al., 2011) and the general negative consequences of relationship conflict such as decreasing levels of team job satisfaction, and performance (e.g., Jehn, 1995; De Dreu and Weingart, 2003), researchers have tried to identify their predictors.

Among antecedents of workplace conflict, individual factors have been most popular among conflict researchers (e.g., Oxenstierna et al., 2011; Danielsson et al., 2015). More recently, various scholars have pointed out the crucial role also played by environmental and organizational factors in the emergence of workplace conflict (e.g., Lu et al., 2011). Aspects of the work environment such as physical work conditions, co-workers’ and supervisors’ social support, or one’s job content (e.g., role ambiguity) have all been shown to relate (either positively or negatively) to workplace conflict’s emergence (e.g., De Raeve et al., 2008). Yet, this area of research still needs to be broadened (see for instance Lu et al., 2011) as empirical studies examining organizational factors remain scarce. What is most interesting about organizational and environmental antecedents is that, in contrast to pure individual factors, they represent factors on which managers and practitioners can more or less directly act upon. Improving physical work conditions, increasing employees’ levels of social support, or wherever possible changing workers’ job content all represent opportunities for leaders and supervisors to experience workplace conflict less often but rather actively manage it. Our aim, in the present research, is thus to further our understanding of workplace conflict antecedents by examining one important organizational variable on which managers have discretionary control, namely perceived organizational support (POS). As POS captures a positive general organizational climate that emphasizes the importance of both employees’ welfare and contributions (Eisenberger and Stinglhamber, 2011), the examination of its links with relationship and task conflict seems particularly relevant.

More precisely, POS is defined as employees’ beliefs concerning the extent to which their organization cares about their well-being and values their contributions (Eisenberger et al., 1986). In the present research, we examine the possibility that POS would reduce relationship conflict and, in contrast, stimulate task conflict. Additionally, this research examines whether the relationship between POS and workplace conflict (i.e., relationship and task conflict) is mediated by employees’ failure-related trust, defined as “the belief that the organization’s actions in case of failure will take into account the employee’s intent to be helpful” (Neves and Eisenberger, 2014, p. 190). In other words, failure-related trust captures how much the employee can trust the organization to act in good faith in case his/her actions would end in failure. Rooted in the *psychological safety* literature, which has been found to be associated to both relationship and task conflict (e.g., Wilkens and London, 2006; Neves and Eisenberger, 2014), this variable seems of particular relevance for our research.

The present research has several contributions. First, it will broaden the POS literature (e.g., Baran et al., 2012; Kurtessis et al., 2017). Although many studies have shown the positive consequences of POS in terms of increasing employees’ positive attitudes toward the organization and work (e.g., Kurtessis et al., 2017), to our knowledge, the influence of POS on workplace conflict has received very limited attention. Therefore, organizational support theory (OST) lacks from a theoretical rationale regarding the relationship between POS and workplace conflict and empirical findings regarding this issue. In addition, this research will contribute to the literature on workplace conflict by examining new organizational factors as potential drivers of workplace conflict. Besides these theoretical contributions, our research has important implications for practitioners and managers because it will provide useful information regarding organizational interventions aiming to manage workplace conflict.

### Workplace Conflict

Individuals often engage in relationship-oriented and task-oriented conflicts (e.g., Jehn, 1995). The former, i.e. relationship conflict, refers to “interpersonal incompatibilities among group members, which typically include tension, animosity, and annoyance among members within a group” (Jehn, 1995, p. 258). Examples of relationship conflicts are conflicts about personal taste, political preferences, values, and interpersonal style (De Dreu et al., 2004). The latter, i.e. task conflict, refers to “disagreements among group members about the content of the tasks being performed, including differences in viewpoints, ideas, and opinions” (Jehn, 1995, pp. 258). Examples of task conflicts are conflicts that arise because of diverging opinions and interests on resource distribution, procedures, or facts interpretations (De Dreu and Weingart, 2003). Broadly speaking then, while task conflicts largely focus on work and task aspects, relationship conflicts relate to non-task, personal issues (Jehn, 2014).

Several scholars and studies have underlined that the distinction between relationship conflict and task conflict is meaningful as they are linked to different consequences (e.g., Jehn, 1995; Amason, 1996; De Dreu and Weingart, 2003). For instance, empirical research has univocally shown the detrimental impact of relationship conflict on work-related and individual outcomes (e.g., Jehn, 1995; De Dreu and Weingart, 2003). Specifically, relationship conflicts reduce the processing ability of teams because individuals composing the group lose their energy arguing with each other rather than solving task-related problems (De Dreu and Weingart, 2003). In line with this, relationship conflict was found to be related to lower levels of innovative behaviors (Lu et al., 2011), to lower levels of team-level job satisfaction (De Dreu and Weingart, 2003) and to higher levels of intentions to quit (e.g., Ismail et al., 2012). Furthermore, relationship conflicts impair performance, an effect that largely emerges in various studies and meta-analyses (De Wit et al., 2012; DeChurch et al., 2013; O’Neill et al., 2013; Ent et al., 2015).

The picture is less straightforward as what concerns task conflicts. On the one hand, several scholars have suggested that task conflict could potentially benefit both individuals and organizations because of the stimulation of divergent ideas and the fostering of greater cognitive understanding of important issues (Jehn, 1994, 1995; De Wit et al., 2012; DeChurch et al., 2013). As such, task conflicts might help increasing group decision quality (e.g., Simons and Peterson, 2000) and team effectiveness performance (e.g., Jehn and Bendersky, 2003). On the other hand, scientists have argued that task conflict could also impeach performance for multiple reasons. First, task conflicts add to employees’ cognitive load and redirect resources away from the task (van Woerkom and Van Engen, 2009). Second, task conflicts have the potential to harm relationships and develop into conflicts that are more relational than task-related. According to Simons and Peterson (2000) for instance, task conflicts are likely to escalate into relationship conflicts because of the use of inappropriate emotional and behavioral reactions that they entail as well as the emergence of cognitive misattributions.

Overall then, the link between task conflicts and performance seems to be quite complex. Research has indeed shown that it varies as a function of moderating situational variables such as task type (Jehn, 1995) or timing (Jehn and Mannix, 2001), and individual characteristics such as trait self-control (Jimmieson et al., 2017) or openness to experience (Loughry and Amason, 2014). Meta-analyses also reflect this complexity by reporting either negative relationships to performance (e.g., De Dreu and Weingart, 2003; O’Neill et al., 2013) or a positive link (DeChurch et al., 2013) at least to the extent that relationship conflicts are controlled for (De Wit et al., 2012).

As indicated above, much is known about the consequences of workplace conflict (e.g., De Dreu and Weingart, 2003; Meier et al., 2013). Earlier research already tried to advance knowledge regarding predictors of workplace conflict. The dominant explanation regarding the emergence of workplace conflict has focused on individual factors that may drive workplace conflict (e.g., Friedman et al., 2000; Oxenstierna et al., 2011; Danielsson et al., 2015). For instance, in a review chapter, De Dreu and Gelfand (2007) distinguished three major sources of workplace conflict: scarce personal resources, the search of maintaining and promoting a positive view of oneself, and the search for validation of one’s opinions and positions. In a different perspective, Halevy et al. (2014) showed across five studies the important relationships that exist between people’s mental models of conflict, their personality and their real-world conflict experiences. More recently, research evidence revealed that organizational factors such as organizational structure (Oxenstierna et al., 2011), leadership (e.g., Neves and Eisenberger, 2014), physical environment (De Raeve et al., 2008), coworkers’ or supervisor’s support, or career advancement opportunities (De Raeve et al., 2008) are all likely to have an influence on workplace conflict. Therefore, “a reasonable assumption is that both individual and organizational factors can explain the emergence of conflicts at the workplace” (Oxenstierna et al., 2011, p. 502). Yet, too few studies in the workplace conflict literature have considered the influence of the organizational context (Lu et al., 2011). In line with this, we thought to examine the influence of POS on the two different types of workplace conflict, i.e., relationship and task conflict. By fostering an organizational climate of respect and positive valuation from the organization and among its members (Eisenberger and Stinglhamber, 2011), POS should indeed be of particular relevance in the (non)emergence of workplace conflict.

### Perceived Organizational Support

POS refers to employees’ perceptions regarding the extent to which their organization thinks highly of their contributions and promotes their welfare (Eisenberger et al., 1986). Relying on the social exchange perspective (Blau, 1964) and the norm of reciprocity (Gouldner, 1960), OST holds that employees feel an inner obligation to reciprocate this favorable and supportive treatment received from their organization by developing favorable attitudes toward the organization and by helping the organization reaching its goals (e.g., Eisenberger et al., 1986). OST also states that POS fulfills employees’ socio-emotional needs (e.g., need for esteem), leading to favorable attitudes and behaviors toward the organization and greater subjective well-being (e.g., Armeli et al., 1998; Kurtessis et al., 2017).

Accordingly, empirical evidence showed that POS is positively related to a plethora of positive attitudes and behaviors at work such as employees’ affective commitment (e.g., Eisenberger et al., 2001), organizational identification (e.g., Sluss et al., 2008), work engagement (e.g., Caesens et al., 2016), and job performance (e.g., Chen et al., 2009; Shoss et al., 2013). POS was also found to be positively related to several indicators of employees’ well-being such as job satisfaction (e.g., Eisenberger et al., 1997; Caesens and Stinglhamber, 2014), and general health (e.g., Bradley and Cartwright, 2002). In contrast, numerous studies indicated that POS decreases employees’ turnover intentions (e.g., Kurtessis et al., 2017), absenteeism (e.g., Eder and Eisenberger, 2008), and burnout (e.g., Kang et al., 2010; Caesens et al., 2017). Interestingly, even if prior studies have thus clearly demonstrated that POS creates a positive environment for employees, the influence of POS on workplace conflict has been ignored.

Yet, POS is likely to be linked to both types of workplace conflict. On the one hand, POS should be negatively associated with relationship conflict because it fosters an organizational climate wherein employees treat each other with respect and are open to a two-way communication (Eisenberger and Stinglhamber, 2011). Further, POS fosters a sense of common identity between members of the organization (Eisenberger and Stinglhamber, 2011). Eisenberger and Stinglhamber (2011) indeed claimed that, when POS is high, employees experience a sense of unity with the organization as a whole. Interestingly, prior research in the literature of conflict reported that “feeling of connection and common identity are important factors for the prevention of interpersonal conflicts” (Oxenstierna et al., 2011, p. 502). For instance, Hinds and Mortensen (2005) claimed that “in the absence of a strong shared identity team members are likely to evaluate other team members’ behaviors negatively assuming a competitive rather than cooperative stance when problems and miscommunication arise” (p. 292). On the contrary, if a common identity is present between group members they will be more prone to be loyal and especially concerned of the well-being of other in-group members (Hinds and Mortensen, 2005). In line with this, Colbert and his colleagues (Colbert et al., 2004) showed that POS is negatively related to employees’ interpersonal deviance behaviors such as saying something hurtful to another employee, making fun of someone at work, or acting rudely toward another coworker. Additionally, whereas it was not the focus of their study, Sulea et al. (2012) reported a significant negative association between POS and interpersonal conflict (*r* = -0.37, *p* < 0.01). Therefore, based on the above reasoning and empirical evidence, we posited that POS would be negatively related to relationship conflict (*Hypothesis 1*).

On the other hand, employees perceiving high support from their organization should be more prone to engage in task conflict and have an open discussion concerning work-related task with their colleagues. OST (e.g., Eisenberger et al., 1986; Rhoades and Eisenberger, 2002; Eisenberger and Stinglhamber, 2011; Kurtessis et al., 2017) states that POS conveys that employees’ contributions are valued and appreciated. Based on the norm of reciprocity (Gouldner, 1960) and the social exchange theory (Blau, 1964), OST further suggests that POS creates among employees a feeling of obligation to reciprocate for the positive treatment received, most notably by aiding the organization to reach its goals. Based on the above, employees might be able to fulfill this embeddedness toward the organization by focusing more on their tasks and by being more prone to share their differences in ideas, opinions, and thoughts related to work issues in order to contribute to a potentially better organizational performance. In other words, the feeling of obligation to return the favorable treatment would stimulate employees to have richer discussions and in-depth investigations on several issues, and to express ideas or recommendations useful to help the organization reaching its objectives, especially since their contributions are welcome and valued by this organization. Second, the group engagement model suggests that a favorable organizational treatment such as a high POS would provide employees with information regarding the organizational respect they benefit from and their high informal status within the organization (Tyler and Blader, 2003). Yet, according to Jetten et al. (2006), people experiencing high status in a group are less sensitive to the social context and susceptible to group influence. As a result, those high-status group members portrayed themselves as more independent and nonconformist than group members with low ingroup status. Therefore, experiencing high POS should lead these high-status employees to share more willingly divergent perspectives and constructive arguments with their colleagues. In line with the two above reasonings, we thus hypothesized that POS is positively related to task conflict (*Hypothesis 2*).

## Study 1: POS and Workplace Conflict

Study 1 was designed to test the first two hypotheses posited in this research, which hold that POS is negatively related to relationship conflict (*Hypothesis 1*) and positively related to task conflict (*Hypothesis 2*).

### Methods

#### Sample and Procedure

The sample consisted of 263 Belgian teachers (53 men, 195 women, and 15 omitted to indicate their gender). All participants spoke French and worked in various primary or high schools of the French-speaking part of Belgium. The average age of participants was 42.24 years (*SD* = 10.96) and their average teaching experience was 15.79 years (*SD* = 10.86). Participants were engaged in teaching activities for an average of 22.00 h per week (*SD* = 4.90 h). The average number of students per school was 782.14 (*SD* = 1895.805).

An email inviting participants to fill in our online questionnaire was sent by a trade union regional secretary to several directors of primary and high schools located in Belgium and affiliated to this union. This email invitation included a brief description of the study objectives and included the URL to access the online questionnaire. It was clearly stipulated in the email invitation and on the first page of the questionnaire that participation was voluntary and that anonymity and confidentiality of the responses provided by participants were guaranteed. Informed consent was obtained by virtue of survey completion. Indeed, it was clearly stated that participants were invited to complete the questionnaire voluntary and freely, and they could stop completion at any time. Further, the school directors were free to decide whether or not to forward the email invitation to teachers working in their school. Because directors did not communicate their decision to us, it was impossible to calculate an exact response rate to the questionnaire. This study was approved by the Ethics Committee of the Psychology Department at the Université catholique de Louvain (Belgium).

#### Measures

##### POS

We measured POS using 8 items of the Survey of POS (SPOS; Eisenberger et al., 1986) [e.g., (Name of the organization/institution) really cares about my well-being”]. In doing so, we followed Rhoades and Eisenberger’s (2002) recommendations to include the two facets of the definition of POS, namely care about employees’ well-being and valuation of employees’ contributions. Participants rated these items using a 7-point Likert-type scale ranging from 1 (“Strongly disagree”) to 7 (“Strongly agree”).

##### Workplace conflict

Relationship and task conflict were measured using a slightly adapted version of the 8-item (i.e., 4 items for relationship conflict and 4 items for task conflict) scale developed by Jehn (1995). More precisely, as it has been done in previous research (e.g., Meier et al., 2013), items were re-worded to reflect positive affirmations, rather than questions as it was the case in the original scale. A sample item for relationship conflict is “*There is friction among members of my organization/institution*” and for task conflict is “*There are differences of opinions regarding work within my organization/institution.*” In order to not artificially inflated the correlation between the two types of conflicts (and thus reduce the common method variance bias; cf. Podsakoff et al., 2003) and in line with what scholar have done in prior research (e.g., Meier et al., 2013), we adapted the format of the response scales associated with these items. For relationship conflict, the response scale ranged from 1 “Not at all” to 7 “Extremely” and, as such, is close to that of Jehn (1995). For task conflict, the response scale ranged from 1 “Never” to 7 “Always” as it has been done by Baillien et al. (2016).

##### Control variables

Following Becker’s (2005) recommendations, the relationships between potential control variables (i.e., gender, age, job tenure as a teacher, number of students in the school -i.e., size of the school-, and weekly hours of teaching) and our dependent variables (i.e., relationship and task conflict) were examined at the empirical level (see Table 1 for more details). None of our potential control variables were correlated with the dependent variables included in our model. Therefore, no control variable was included in subsequent analyses (see Becker, 2005).

**Table 1:** Study 1: Descriptive statistics and intercorrelations among variables.

Variable	*M*	*SD*	1	2	3	4	5	6	7	8
(1) Gender	–	–	–	0.14*	0.01	0.01	0.01	0.05	–0.02	0.03
(2) Age	42.24	10.96		–	0.76***	0.13*	–0.14*	–0.11	0.02	0.05
(3) Job tenure as teacher	15.79	10.86			–	0.12	–0.07	–0.05	–0.05	–0.01
(4) Number of students in the school	782.14	1895.805				–	0.05	–0.06	0.09	0.12
(5) Weekly hours of teaching	22.00	4.90					–	0.03	0.08	0.12
(6) POS	3.61	1.45						(0.91)	–0.43***	–0.42***
(7) Relationship conflict	4.07	1.48							(0.94)	0.68***
(8) Task conflict	4.05	1.02								(0.81)


*N = 263 (excepted for gender *N* = 248, age *N* = 250, job tenure *N* = 251, number of students in the school *N* = 244, weekly hours of teaching *N* = 248). α coefficients are reported on the diagonal. POS = perceived organizational support. Females were coded 0 and Males were coded 1. ^∗^*p* < 0.05, ^∗∗^*p* < 0.01, and ^∗∗∗^*p* < 0.001.*

### Results

#### Discriminant and Convergent Validity

To examine whether the variables presented in our model were distinct constructs (i.e., POS, relationship conflict, and task conflict), we performed confirmatory factor analyses using the LISREL 8.8 software (Jöreskog and Sörbom, 1993). Results of these analyses indicated that the hypothesized three-factor model fitted the data well [χ^2^(101) = 283.04, CFI = 0.97, NNFI = 0.97, RMSEA = 0.08]. Using chi-square difference tests (Bentler and Bonett, 1980), we compared its fit to the fit of several nested models (i.e., two-factor and one-factor models). These analyses indicated that the three-factor model was superior to all more constrained models. Additionally, all items loaded reliably on their respective latent constructs with standardized loadings ranging from 0.55 to 0.88 for POS, from 0.76 to 0.96 for relationship conflict, and from 0.45 to 0.92 for task conflict.

#### Relationships Among Variables

Table 1 displays the means, standard deviations, internal reliabilities, and intercorrelations among variables. As firstly hypothesized, POS was negatively associated with relationship conflict. However, contrary to our second hypothesis, POS was also negatively associated with task conflict.

#### Test of Hypotheses

Using Lisrel 8.8 (Jöreskog and Sörbom, 1993), we conducted structural equation modeling to test our two hypotheses in a single structural model. Because the correlation between relationship conflict and task conflict was relatively high in our sample (see Table 1), the disturbance terms associated with these two variables were allowed to freely correlate. Results indicated that the structural model fitted the data very well [χ^2^(101) = 283.04, CFI = 0.97, NNFI = 0.97, RMSEA = 0.08]. Standardized estimates indicated that POS is negatively associated with both relationship (γ = -0.48 *p* < 0.001) and task conflict (γ = -0.46, *p* < 0.001), supporting Hypothesis 1 and failing to support Hypothesis 2.

### Discussion

Study 1’s findings indicated that, as expected, POS is negatively related to relationship conflict. This result suggests that POS fosters a climate of respect and a sense of common identity among employees that is not conducive to relational conflict. Contrary to our second hypothesis, we did not find support for the positive influence of POS on task conflict among employees. Our results indicated that POS influences task conflict in the opposite direction such as it decreases rather than stimulates task conflict. This suggests that when POS is high, employees are less prone to challenge ideas and positions with other members of the organization, which could lead to premature agreement regarding a work-related topic or to miss alternative innovative solutions. This unexpected negative relationship found between POS and task conflict might be explained by the fact that POS increases not only social cohesion and a common identity between members but also the cohesion regarding how tasks should be performed at work. POS, through its actions might elicit a unified culture of respect, appreciation and mutual caring between all organizational members (Eisenberger and Stinglhamber, 2011). This unified culture might lessen task conflict such as having frequent and intense divergence in opinions, ideas, and thoughts between organizational members.

Despite its contributions to the organizational support and workplace conflict literatures, Study 1 also presents important limitations. First, results of this study might be restricted to a specific population of workers, i.e., teachers. Given the particular population assessed in our study, more data are needed in order to generalize our findings to other organizational contexts. Second, no attention was given to potential mechanisms underlying the relationship between POS and workplace conflict. In order to overcome these limitations, in Study 2, we attempt to replicate these findings among another population of workers. More importantly, we examine a potential mediator of the relationship between POS and workplace conflict. Examining a mediator of this relationship will help to better understand the results obtained in our Study 1 and, more particularly, it will bring a more nuanced view of the unexpected negative relation found between POS and task conflict.

More precisely, we argue that how much employees perceive their organization as a safe place to discuss problems, tough issues, or even mistakes they made should explain the relationships between POS and workplace conflict. Close to this idea is the psychological safety concept defined as the “shared belief that the team is safe for interpersonal risk taking” (Edmondson, 1999, p. 354) and referring to a “sense of confidence that the team will not embarrass, reject, or punish someone for speaking up.” While focusing on the *team* level and not on the *organizational* target, the literature on psychological safety is very instructive for the present research. This literature already indicated that psychological safety might help to reduce relationship conflict in a team. Bradley et al. (2012, p. 151) indeed claimed that, “psychological safety may amplify the involvement of each team member and the intensity of interaction among teammates without endangering the harmony of the team.” In line with this perspective, prior research reported negative correlations between psychological safety and relationship conflict (Wilkens and London, 2006; Martins et al., 2013).

While findings on the relationship between psychological safety and relationship conflict are thus quite clear in this literature, the relationship between psychological safety and task conflict seems much more uncertain. On the one hand, individuals in a high psychological safety climate should be more likely to ask for help or to share an idea or opinion even if they are not correct on this issue (Edmondson, 1999; Wilkens and London, 2006). Accordingly, the relationship between psychological safety and task conflict might be positive. On the other hand, in teams where a certain psychological safety is experienced, employees should be less likely to engage in task-related conflictual discussions because of the unified and secure atmosphere. Yet, as indicated by Greer et al. (2007, p. 7), “without such critique or debate, task conflict will not exist within teams.” Accordingly, the relationship between psychological safety and task conflict might be negative. In line with this second possibility, prior empirical research indeed reported negative correlations between employees’ psychological safety and task conflict (Wilkens and London, 2006; Bradley et al., 2012).

As previously stated, the psychological safety concept refers to the *team* level. Some authors have however considered that this concept might also make sense at the organizational level. Precisely, applying it to the organizational target, Neves and Eisenberger (2014) proposed the failure-related trust construct defined “as the belief that the organization’s actions in case of failure will take into account the employee’s intent to be helpful” (Neves and Eisenberger, 2014, p. 190). In other words, employees who perceive a high level of failure-related trust in their organization are not afraid to put themselves in a situation of vulnerability or to discuss of difficulties they might have at work (Neves and Eisenberger, 2014), failure-related trust should be related to workplace conflict. More precisely, failure-related trust should be negatively related to relationship conflict whereas the link between failure-related trust and task conflict may be either positive or negative. On the one hand, when employees perceive high level of failure-related trust in their organization, they should be more willing to put themselves in a situation of vulnerability and thus will be more likely to express challenging ideas without fearing negative consequences. On the other hand, when failure-related trust is high, members of the organization might be less prone to engage in real conflictual forms of task-related discussions because of the unified and secure atmosphere. As a whole, failure-related trust should thus be related to both types of workplace conflict.

Moreover, according to Neves and Eisenberger (2014), POS fosters the perception of failure-related trust among employees. Employees perceiving high levels of organizational support are more prone to believe that their inputs and competences are valued and that their mistakes will be received with leniency (Neves and Eisenberger, 2014). At the empirical level, Neves and Eisenberger (2014) showed that POS is, indeed, positively related to employees’ failure-related trust, which, in turn, increased employees’ propensity to take risks. Taking together, we thus hypothesized that failure-related trust mediates the negative relationship between POS and relationship conflict (*Hypothesis 3a*). Furthermore, we also hypothesized that failure-related trust mediates the negative relationship between POS and task conflict (*Hypothesis 3b*).

## Study 2: The Mediating Role of Failure-Related Trust in the Relationship Between POS and Workplace Conflict

Study 2 was first designed to replicate results of Study 1 among another population than teachers. Furthermore, Study 2 aimed to test Hypotheses 3a and 3b, which hold that failure-related trust (Neves and Eisenberger, 2014) acts as a mediator in the relationship between POS and workplace conflict (i.e., both relationship and task conflict).

### Methods

#### Sample and Procedure

Employees from a Belgian organization specialized in employment and training services were contacted via an email in order to complete an electronic questionnaire that was part of a larger survey. Informed consent was obtained by virtue of survey completion. Indeed, it was clearly stated in the first page of the questionnaire that participants were invited to complete the questionnaire voluntary and freely and they could stop completion at any time. Further, the confidentiality and anonymity of the participants were assured. This study was approved by the Ethics Committee of the Psychology Department at the Université catholique de Louvain (Belgium). In total, 692 employees accepted to respond to the questionnaire (response rate = 34.72%). Of these 692 participants, only 477 fully completed the questionnaire on our variables of interest (i.e. POS, failure-related trust, relationship and task conflict) and thus composed the final sample. Of this sample, 71.07% were women, 18.45% men and 10.48% omitted to indicate their gender. Furthermore, the average age of this sample was 43.00 years (*SD* = 8.83) and participants were working for this organization for an average of 12.21 years (*SD* = 8.73). Regarding their level of education, most of participants held a bachelor degree (39.20%).

#### Measures

##### POS

We used the same scale as in Study 1 to measure perceptions of organizational support.

##### Failure-related trust

Failure-related trust was measured using the 4-item scale from Neves and Eisenberger (2014). These four items “refer to how safe employees feel in the case of failure, demonstrated by actions such as discussing problems and mistakes” (Neves and Eisenberger, 2014, p. 194). These items are “*If I had difficulties at work, I would be inclined to keep them from my organization*” (reversed coded), “*I would feel comfortable telling my organization about a mistake I made*,” *When I am not good at a task, I feel at ease telling my organization about it*” and “*If I had a problem that could influence my performance at work, I would hesitate to discuss it with my organization*” (reversed coded). Furthermore, due to a low internal reliability coefficient obtained in this prior study (i.e., α = 0.60) (Neves and Eisenberger, 2014), we decided to add two adapted items from the psychological safety scale of Edmondson (1999) replacing “the team” by “my organization.” These two items were “*My organization holds against me when I make a mistake*” and “*In my organization, it is easy to bring up problems and tough issues.*” Participants responded to these statements using a 7-point Likert-type scale, with response options ranging from 1 (“Strongly disagree”) to 7 (“Strongly agree”).

##### Workplace conflict

The workplace conflict scale used in Study 1 was also used in this second study.

##### Control variables

As in Study 1, we analyzed the empirical relationships between potential control variables (i.e., socio-demographic variables) and the dependent variables included in our model (i.e., failure-related trust and workplace conflict). As showed in Table 2, level of education was found to display a significant correlation with failure-related trust (*r* = -0.10, *p* < 0.05), whereas gender (*r* = 0.11, *p* < 0.05) and organizational tenure displayed (*r* = 0.11, *p* < 0.05) significant correlations with relationship conflict. In addition, age (*r* = 0.13, *p* < 0.01), organizational tenure (*r* = 0.15, *p* < 0.01), and level of education (*r* = 0.11, *p* < 0.05) reported positive correlations with task conflict. We decided to perform our analyses with and without these control variables as recommended by Becker (2005) and Becker et al. (2015)^1^. The pattern of results was essentially identical and did not change the interpretation of the findings. Therefore, we decided to report our results without control variables in order to reduce model complexity (Becker, 2005).

**Table 2:** Study 2: Descriptive statistics and intercorrelations among variables.

Variable	*M*	*SD*	1	2	3	4	5	6	7	8
(1) Gender	–	–	–	–0.15**	–0.10*	0.03	–0.04	–0.04	0.11*	0.01
(2) Age	43.00	8.83		–	0.65***	–0.07	–0.16**	–0.02	0.03	0.13**
(3) Organizational tenure	12.21	8.73			– –	–0.14**	–0.23***	–0.05	0.11*	0.15**
(4) Level of education	–	–				–	–0.03	–0.10*	0.05	0.11*
(5) POS	3.61	1.16					(0.87)	0.45***	–0.34***	–0.37***
(6) Failure-related trust	4.69	1.09						(0.75)	–0.31***	–0.29***
(7) Relationship conflict	3.95	1.38							(0.94)	0.63***
(8) Task conflict	4.02	1.02								(0.86)


*N = 477 (excepted for gender *N* = 427, age *N* = 421, organizational tenure *N* = 424, and education *N* = 427). α coefficients are reported on the diagonal. POS = perceived organizational support. Females were coded 2 and Males were coded 1. ^∗^*p* < 0.05, ^∗∗^*p* < 0.01, and ^∗∗∗^*p* < 0.001.*

### Results

#### Discriminant and Convergent Validity

To assess the discriminant validity between POS, failure-related trust, relationship conflict, and task conflict, we compared several nested models using Lisrel 8.8 (Jöreskog and Sörbom, 1993). Results of these analyses indicated that the fit indices for the hypothesized four-factor model were satisfactory [χ^2^(203) = 746.51; RMSEA = 0.07*;* CFI = 0.96; NNFI = 0.96] and significantly superior to that of all more constrained models (i.e., three-factor, two-factor, and one-factor models). Furthermore, each item loaded reliably on its latent construct with standardized loadings ranging from 0.46 to 0.80 for POS, from 0.49 to 0.67 for failure-related trust, from 0.78 to 0.96 for relationship conflict, and from 0.56 to 0.91 for task conflict.

#### Relationships Among Variables

Descriptive statistics, reliability coefficients, and intercorrelations among variables are presented in Table 2. As expected, POS was positively related to failure-related trust, and POS and failure-related trust were both negatively associated with relationship conflict and task conflict.

#### Test of Hypotheses

**FIGURE 1:**
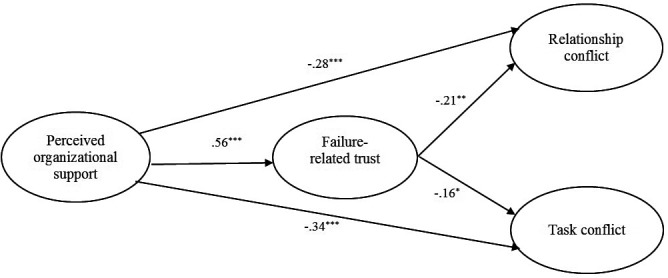
Study 2: completely standardized path coefficients for the alternative model 2. *N* = 477. *^∗^p* < 0.05, *^∗∗^p* < 0.01, and ^∗∗∗^*p* < 0.001.

The hypothesized structural relationships among latent variables were assessed using the structural equation modeling approach. As in Study 1, due to a high correlation between the two forms of workplace conflict, we allowed the disturbances terms of relationship and task conflict to correlate. We conducted a preliminary analysis in order to assess the direct effect of POS on both relationship conflict and task conflict. Results indicated that this model fitted the data accurately [χ^2^(101) = 372.18, CFI = 0.98, NNFI = 0.97, RMSEA = 0.08]. In addition, standardized estimates indicated that POS is negatively related to both relationship (γ = -0.39, *p* < 0.001) and task conflict (γ = -0.42, *p* < 0.001), yielding similar results than those obtained in Study 1. We then tested our hypothesized model wherein failure-related trust would act as a full mediator of these relationships. The results indicated that this model fitted the data accurately [χ^2^(205) = 785.09, CFI = 0.96, NNFI = 0.95, RMSEA = 0.08]. Nevertheless, based on the chi-square difference tests (Bentler and Bonett, 1980), an alternative model (i.e., alternative model (1) adding a direct path between POS and relationship conflict was statistically superior to the hypothesized model [Δχ^2^(1) = 6.25, *p* < 0.05]. In addition, adding a path between POS and task conflict (i.e., alternative model (2) provided a better fit than that of the alternative model 1 [Δχ^2^(1) = 32.33, *p* < 0.001]. Consequently, the alternative model 2 was retained as the best depiction of the data [χ^2^(203) = 746.51, CFI = 0.96, NNFI = 0.96, RMSEA = 0.08]. Figure 1 displays the standardized parameter estimates for this alternative model 2. As it can be seen, POS is positively related to failure-related trust (γ = 0.56, *p* < 0.001), which, in turn, has a significant negative influence on both relationship conflict (β = -0.21, *p* < 0.01) and task conflict (β = -0.16, *p* < 0.05). Furthermore, results indicate that POS is directly and negatively related to relationship conflict (γ = -0.28, *p* < 0.001) and task conflict (γ = -0.34, *p* < 0.001). Finally, as recommended by Hayes (2013), we tested for the indirect effects using the PROCESS macro (model 4) for SPSS to obtain the bootstrapped confidence intervals. The results of these additional analyses indicate that the indirect effects of POS on relationship and task conflict via failure-related trust are significant (*b* = -0.1033, BCa 95% CI = [-0.1683; -0.0488]; *b* = -0.0618, BCa 95% CI = [-0.1063; -0.0235]), supporting Hypotheses 3a and 3b. As POS remains directly related to both types of conflicts even when failure-related trust is controlled for, the indirect effects are moderate, and we can conclude to a partial mediating effect of failure-related trust.

### Discussion

Consistent with findings of the first study, Study 2 showed that POS is negatively associated with both types of workplace conflict, namely relationship and task conflict. More importantly, besides replicating results of Study 1, this second study aimed to investigate a potential underlying mechanism of the negative relationship between POS and workplace conflict. Extending prior findings from Neves and Eisenberger (2014), we showed that a higher level of POS leads to a higher level of failure-related trust, which, in turn, reduces both relationship and task conflict. Precisely, results indicated that employee’ failure-related trust acts as a partial mediator in the negative relationship between POS and the two types workplace conflict (i.e., relationship and task conflict).

## General Discussion

The main aim of the present research was to examine the relationship between POS and workplace conflict. More precisely, the purpose of this research was to examine whether POS would reduce relationship conflict and stimulate task conflict (Study 1-2). Additionally, this research aimed to study whether failure-related trust acts as a key mediator in the relationship between POS and workplace conflict (Study 2).

Firstly, our results indicated across two studies that, as expected (Hypothesis 1), POS is negatively related to relationship conflict and, contrary to our second hypothesis, also negatively linked to task conflict. The negative relationship found between POS and relationship conflict is in line with the proposition that POS induces a sense of common identity between organizational members (Eisenberger and Stinglhamber, 2011) that helps to lessen relationship conflict (e.g., Hinds and Mortensen, 2005). Furthermore, this finding is consistent with Colbert et al. (2004) results, which revealed that POS has a negative influence on employees’ interpersonal deviance such as acting rudely toward another coworker. These results also replicate the negative correlation found between POS and relationship conflict as reported in Sulea et al. (2012) study.

Secondly, the negative association found between POS and task conflict in Study 1 and 2 suggests that POS also leads to higher cohesion regarding how work and tasks should be carried. POS, through its actions, elicits a unified culture of respect and appreciation among organizational members (Eisenberger and Stinglhamber, 2011) that is not conducive to task conflict. Because task conflicts potentially stimulate critical-thinking, this negative association found between POS and task conflict in Study 1 and 2 would suggest a potential negative side of the POS construct. This result, indeed, suggests that employees who perceive high levels of organizational support are more likely to prematurely agree on a topic without sufficient discussion. That is, POS could lead to an unawareness of relevant alternative solutions or point of views that could benefit the whole organization and/or stimulate innovative solutions. Indeed, scholars have argued that task conflict is likely to improve the use of debate within a team and the subsequent decision quality (Amason, 1996). Lu et al. (2011) also showed that task conflict has positive consequences for organizations such as increasing knowledge sharing behaviors and innovative behaviors and positive effects of task conflicts on performance have been evidenced in some meta-analytic reviews (e.g., De Wit et al., 2012; DeChurch et al., 2013).

However, reducing task conflict could also present some benefits. Prior research on the consequences of task conflict is still not completely conclusive regarding its influence on employees’ performance. That is, although some meta-analytic results suggest a host of positive consequences emerging from task conflicts (DeChurch et al., 2013), other studies indicate that the relationship between task conflict and performance could also be overly negative (De Dreu and Weingart, 2003; O’Neill et al., 2013) or could depend on the simultaneous presence of relationship conflict (De Wit et al., 2012). These mixed results have led scholars to the conclusion that that the exact effects of task conflict on performance might depend on moderating variables linked to the situation (e.g., type of task, Jehn, 1995) or the individual (e.g., openness to experience, Loughry and Amason, 2014). Similarly, several authors have suggested that task conflict could negatively affect individuals’ well-being and attitudes through the increase of relationship conflict that it entails (e.g., Medina et al., 2005) although the escalation of task conflict into relationship conflict seems, in fact, to also depend on people’s individual traits (Jimmieson et al., 2017). As a whole, these results suggest that the reduction of task conflict that is observed when POS is high could also be beneficial, particularly in terms of employees’ subjective health and well-being.

The negative association found in this research between POS and task conflict, might arise because a high level of POS leads to higher levels of cooperation among employees. Individuals working in an environment characterized by cooperativeness might be more prone to engage in behaviors that lead to a constructive process of conflict resolution (Deutsch, 2013). Consequently, it is possible that employees effectively share different opinions and arguments toward a specific work-related topic but that these ideas and divergent opinions are shared within a climate of respect and appreciation. Therefore, these discussions and divergent opinions are not remembered as a conflict by employees when directly asked. In line with this idea, prior scholars have underlined that self-reported measures of workplace conflict are subjected to memory bias (e.g., Meier et al., 2013). For instance, Meier et al. (2013) suggested that the best way to assess workplace conflict and to capture its experience is to directly ask participants to report a conflict event immediately after its occurrence (e.g., Campbell and Graziano, 2002). In the same vein, it is possible that POS increases knowledge sharing or stimulates divergent ideas generation among employees. In line with this view, Kerwin et al. (2011) found, in their qualitative study conducted among a sample of nonprofit board members, that their participants experienced and reported “differences of opinions,” “debates” or “professional discussion” but not “conflict” *stricto sensu* within their organizations. Accordingly, they perceived the so-called conflict as functional for decision quality and idea generation. In line with this, because of the measure we used in the present research, we could not directly capture this positive view of “conflict.” Therefore, future research should examine whether POS is able to foster knowledge sharing and divergent ideas generation among members with more appropriate measures.

Our research also provides further insight regarding the negative relationship found between POS and task conflict by examining a mediator of the relationship between POS and workplace conflict, namely failure-related trust. Results of Study 2 indeed indicated that the negative relationship found between POS and each type of workplace conflict is partially mediated by employees’ failure-related trust. The more employees perceive that their organization values their contributions and cares about their well-being, the more they feel that their organization will be willing to respond compassionately to the employees’ desire to share difficulties at work. This perception of failure-related trust, in turn, reduced both relationship and task conflict. These results are also consistent with prior research, which reported a negative relationship between psychological safety and relationship conflict (e.g., Wilkens and London, 2006), and between psychological safety and task conflict (e.g., Wilkens and London, 2006; Martins et al., 2013). These negative associations found between failure-related trust and both types of workplace conflict are in line with the idea that psychological safety or failure-related trust are context-shifting states that can alter employees’ interactions – and their perceptions of them – with their colleagues within the organization. As described by Bradley et al. (2012), psychological safety or failure-related trust would create a work environment that is not perceived as threatening and, as such, would amplify the involvement of employees and intensify their interactions without endangering the harmony among them. In such an environment, dysfunctional interactions among employees are discouraged so that frustration and hurt feelings are automatically reduced. In contrast, divergent ideas, innovative suggestions and new viewpoints are more than permitted: they are encouraged, without damaging interpersonal interactions (Bradley et al., 2012). Accordingly, when employee’s perceived high psychological safety or trust from their organization in case of failure, they will be less likely to engage in real conflictual forms of task conflict or, as suggested above, to even perceive “conflict” behind a divergence of opinion (Kerwin et al., 2011). This result is in line with the arguments from Greer et al. (2007) who claimed that “without such critique or debate, task conflict will not exist within teams” (p. 7).

Overall, our studies contribute to both the literatures on POS and on workplace conflict. First, our results extend the findings from Neves and Eisenberger (2014) by showing that the POS – failure-related trust association is able to decrease workplace conflict (both relationship and task conflict). As such, these findings contribute to an advancement of OST (e.g., Eisenberger and Stinglhamber, 2011), particularly by showing that failure-related trust is an important mechanism in the relationship between POS and its attitudinal consequences (Neves and Eisenberger, 2014). Second, with regard to the literature on workplace conflict, our research emphasizes, with other recent empirical endeavors (e.g., Oxenstierna et al., 2011), that scholars should take into account the influence of organizational factors while examining workplace conflict’s antecedents. This was so far a rather unexplored issue in the conflict literature, while most scholars have examined individuals’ factors that drive workplace conflict (e.g., Danielsson et al., 2015).

### Limitations and Future Research

Several limitations should be acknowledged while interpreting the results found in this research. First, results of our research are based on cross-sectional designs. Therefore, caution is needed regarding the direction of causality between the variables included in our studies. We cannot exclude the possibility of a reverse causality among our variables. For instance, it is possible that relationship conflict leads to lower levels of POS because experiencing frequent conflict with colleagues might convey the perception among employees that their organization cares too little about the quality of their workplace environment and thus their well-being. Therefore, future research using designs with repeated measurements is strongly needed to address this interesting question. In the same vein, while our theoretical rationale building on OST led us to propose that failure-related trust mediates the relationships between POS and workplace conflict, our design does not allow us to confer this definitive status to this variable. We cannot exclude that failure-related trust may also or rather act as a moderator of the relationships between workplace conflict and its determinants or outcomes. Such a possibility would be in line with Bradley et al. (2012) findings that psychological safety moderates the task conflict – performance relationship.

Second, our research is based exclusively on self-reported measures, which raise important concerns regarding the common method variance bias. Nevertheless, as recommended by Podsakoff et al. (2003), we assured respondents for the anonymity and confidentiality of their honest responses. Furthermore, we conducted statistical analyses as recommended by Podsakoff et al. (2003) to assess this potential threat a posteriori. The Harman’s single-factor test was performed and results revealed a very poor fit of the one-factor model structure in both studies [i.e., χ^2^(104) = 2415.18, CFI = 0.79, NNFI = 0.76, and RMSEA = 0.29 in Study 1 and χ^2^(206) = 4925.81; CFI = 0.78; NNFI = 0.76 and RMSEA = 0.22 in Study 2]. Thanks to these precautions and statistical evidence, we are quite confident that our results were not highly affected by the common method variance problem.

Third, our reliance on two samples of Belgian workers made it hard to assess the extent to which our results would generalize to more general populations of workers. Our demonstration that the observed results generalize among workers coming from two different professional fields (i.e., teaching versus employment and training) somehow alleviate these concerns. Still, it remains important for future research to rely on samples of workers coming from various organizations and industries.

Fourth, the measure we used to assess workplace conflict did not differentiate conflicts with the supervisor or with coworkers. However, prior scholars (Frone, 2000) have stressed that the consequences of workplace conflict might differ depending on the target with whom the conflict is experienced. Furthermore, the way we measured workplace conflicts does not specifically address whether conflicts occur at the team or the individual level (e.g., Lu et al., 2011) nor does it actually assess participants’ own personal experience of conflict. It would be interesting for future research to replicate our model using a more specific operationalization of conflict such as, for instance, relationship conflict with the direct supervisor or with coworkers, and to assess workers’ individual experience of conflict rather than their perceptions of the general conflict climate in the organization.

Finally, our results indicated that, beyond the indirect effect of POS on workplace conflict through failure-related trust, a direct relationship between POS and workplace conflict still occurs. This suggests that other mechanisms are likely at play to explain the dynamic of the relationship between POS and workplace conflict. It would be interesting for future research to look at these other mechanisms that might explain how POS relates to both types of workplace conflict. In addition, it is also possible that contextual or individual factors moderate the relationship between POS and workplace conflict. For instance, the positive consequences of POS in terms of reducing relationship conflict might be exacerbated among individuals who strongly endorse the exchange ideology norm referring to “employees’ belief that it is appropriate and useful to base their concern with the organization’s welfare and their work effort on how favorably they have been treated by the organization” (Eisenberger et al., 2001, pp. 42–43). Accordingly, employees who strongly endorse the exchange ideology should be more sensitive to reciprocate the high level of POS (Eisenberger et al., 2001) by abiding organizational standards related to interpersonal relationships (Colbert et al., 2004). Therefore, the negative association between POS and relationship conflict will be stronger for these individuals. In the same vein, we could expect that the relationship between POS and task conflict is positive rather than negative under certain circumstances. More precisely, organizational factors such as an innovative organizational culture that, by definition, is characterized by creativity and risk taking (Taormina, 2009) should be a necessary context to foster task conflict. In line with this, POS might interact with a high innovative culture, so that POS would be positively related to task conflict in a receptive organizational context characterized by a high innovative culture. On the contrary, in a low innovative context as our samples seem to be characterized, POS would fail to stimulate task conflict among organizational members. Consequently, a general promising and challenging step for future research is to investigate key factors that moderate the relationship between POS and workplace conflict.

### Practical Implications

This research has important implications for managers because it identifies ways to manage workplace conflict. Because relationship conflict was found to have a deleterious impact on employees’ performance (Lu et al., 2011), decision quality (Amason, 1996) and well-being (e.g., Sonnentag et al., 2013) and because the consequences of task conflict are not clear, interventions should be aimed to reduce workplace conflict. In line with this idea, the results of our research suggest that employees perceiving high levels of support from their organization will experience lower levels of relationship conflict and less task conflict.

OST (Eisenberger et al., 1986; Eisenberger and Stinglhamber, 2011) suggests two important ways to increase employees’ perceptions of organizational support. First, managers should adopt procedures and human resources practices that increase employees’ perceptions of organizational support. Prior studies (e.g., Rhoades and Eisenberger, 2002) have indicated that perceptions of organizational support could be foster by providing fairness among decisional policies, maintaining open channels of communication with their employees, assuring employees that their jobs are secure, offering valuable training or developmental programs that promote employees’ personal growth, and/or eliminating continual work overloads (Eisenberger and Stinglhamber, 2011). Second, POS is influenced by the employee’s perception of the support received from his/her supervisor (e.g., Rhoades and Eisenberger, 2002; Kurtessis et al., 2017). Therefore, several scholars suggested that organizations should encourage managers to be supportive to their subordinates, for instance by having regular meetings with their subordinates, resolving any conflicting job responsibilities, or providing subordinates with the materials or emotional resources they need (e.g., Eisenberger and Stinglhamber, 2011; Newman et al., 2012). More recently, Gonzalez-Morales et al. (2016) developed and provided empirical evidence for the efficacy of a brief supervisor support training program including four basic strategies, i.e., benevolence, sincerity, fairness, and experiential processing.

## Conclusion

The present research examined for the first time the influence of POS on workplace conflict. Results of two studies indicated that POS is negatively related to both relationship and task conflict and that failure-related trust partially mediates these associations. As noted above, understanding the organizational antecedents of workplace conflict is essential as it is easier to modify and act on the organizational context than on individual variables. Yet, not all organizational factors can be acted upon. Changing a job’s content or the physical work environment, for instance, might represent interesting yet practically unfeasible avenues for reducing workplace conflict. In contrast, we already know a lot about the means by which employees’ level of POS can be increased (e.g., Rhoades and Eisenberger, 2002; Eisenberger and Stinglhamber, 2011; Kurtessis et al., 2017). Thus, on top of increasing our understanding of the antecedents of workplace conflict, the present research offers a practical tool to prevent its emergence in organizations.

## Author Contributions

GC, FS, and SD conceived and designed the experiments. GC, MDW, and AM performed the experiments and data collection. GC analyzed the data. GC, FS, and SD wrote the paper. All authors contributed to manuscript revision, read, and approved the submitted version.

## Conflict of Interest Statement

The authors declare that the research was conducted in the absence of any commercial or financial relationships that could be construed as a potential conflict of interest.

## Footnotes

1 Missing values of the socio-demographic variables were computed using the multiple imputation method in Lisrel.
